# ‘Why don’t you just use a condom?’: Understanding the motivational tensions in the minds of South African women 

**DOI:** 10.4102/phcfm.v2i1.79

**Published:** 2010-05-07

**Authors:** Rachel Mash, Bob Mash, Pierre de Villiers

**Affiliations:** 1 Fikelela AIDS Project Anglican Diocese of Cape Town, South Africa; 2 Division of Family Medicine and Primary Care, Stellenbosch University, South Africa

## Abstract

**Background:**

HIV/AIDS makes the largest contribution to the burden of disease in South Africa and consistent condom use is considered a key component of HIV-prevention efforts. Health workers see condoms as a straightforward technical solution to prevent transmission of the disease and are often frustrated when their simple advice is not followed.

**Objectives:**

To better understand the complexity of the decision that women must make when they are asked to negotiate condom use with their partner.

**Method:**

A literature review.

**Results:**

A key theme that emerged included unequal power in sexual decision making, with men dominating and women being disempowered. Women may want to please their partner, who might believe that condoms will reduce sexual pleasure. The use of condoms was associated with a perceived lack of ‘real’ love, intimacy and trust. Other factors included the fear of losing one’s reputation, being seen as ‘loose’ and of violence or rejection by one’s partner. For many women, condom usage was forbidden by their religious beliefs. The article presents a conceptual framework to make sense of the motivational dilemma in the mind of a woman who is asked to use a condom.

**Conclusion:**

Understanding this ambivalence, respecting it and helping women to resolve it may be more helpful than simply telling women to use a condom. A prevention worker who fails to recognise this dilemma and instructs women to ‘simply’ use a condom, may well encounter resistance.

## INTRODUCTION

Sub-Saharan Africa has the highest number of people infected with HIV. Women in Africa are becoming infected in higher numbers than men and it is widely acknowledged that young women, aged 15–24 years, constitute a particularly important group for targeting HIV prevention.^1^

The use of male condoms is seen as one of the most important components of a risk-reduction strategy for HIV. However, although condoms may be highly efficacious and HIV transmission interrupted in as many as 99% of encounters, they cannot be effective as a strategy if they are not utilised.

According to the first South African national youth risk-behaviour survey, only 40% of male and 31% of female adolescents always use a condom.^2^ These figures are similar to national surveys in Burkina Faso, Ghana, Malawi and Uganda, which show male adolescent condoms use during the last sexual encounter to be between 39% and 51%, while females’ use was between 24% and 38%. ^3^ It therefore appears that there is a tendency for women to use condoms less consistently than men.

 Educating patients on the use of condoms can be a frustrating experience for health workers. On the one hand, condoms have the attraction of being a relatively straightforward, efficacious and technical solution to the problem of HIV transmission. On the other hand, patients are often passively disinterested or non-compliant with the message that they should use a condom. Evidently, the knowledge that condoms prevent HIV transmission does not in itself always lead to action and the decision to use a condom is more complex than the simple health education message implies.

Women are often the recipients of this educational message when they go for family planning, antenatal care or seek treatment for sexually transmitted infections (STIs). However, unless they intend using female condoms, which are difficult to obtain, they can only use condoms if they first negotiate condom use with their partner. 

This article explores this scenario and attempts to understand the motivational dilemma in the mind of a woman who is asked to use a condom. Understanding the likely ambivalence and helping women to resolve it may be more helpful than simply telling women to use a condom.

A literature search was conducted on Medline and Google Scholar using the key words AIDS, Africa, gender and condom use. The following themes emerged from the literature and are later summarised in a conceptual framework illustrating the motivational dilemma.

## DISEMPOWERMENT

Using or not using a condom is not simply a question of safer sexual behaviour; it is the outcome of a negotiation between potentially unequal partners. Condoms are not neutral objects about which a straightforward decision can be made on health grounds. Sexual encounters may be sites of struggle between the exercise and acceptance of male power, male definitions of sexuality and women’s ambivalence and resistance. ^4^ A key problem is that the condom message calls upon the woman to assert dominance in the sexual act. Almost everywhere such dominance is not their traditional role and imposes unfamiliar behaviour on both members of the couple.^5^ Condom use increases when power is shared more equitably in the relationship and decreases when women have little power or control. ^6^ Adolescent girls in KwaZulu–Natal used a condom when this was initiated by a man but felt it would be easier to refuse sex completely than to negotiate condom use themselves. Decisions to use condoms were controlled by males, with the tacit agreement of their female partners.^7^

Gender-based power inequalities generally incorporate the belief that men should control women’s sexuality and childbearing capacity. If women practice family planning then the male partner loses this control. Concerns expressed by men include the fear that they will lose their role as head of the family, that their partners will become promiscuous or adulterous and that they will be ridiculed by other people. ^6^ Therefore, in a relationship of unequal power, it becomes very difficult for a woman to negotiate the use of condoms.

## PLEASING YOUR PARTNER

Men are more likely to believe that using a condom will lessen sexual pleasure, diminish intimacy, waste sperm, be like masturbation or be associated with a loss of virility. ^8^ In Jewkes’ study of women from three South African provinces, although 39% of couples had discussed HIV and in almost one-third of relationships women had suggested using condoms, 36% of men said they did not like them and 2% of men accused the woman of infidelity. ^9^ In Varga’s study of youth from Durban, among women who had discussed condoms, 42% said their partners usually refused condoms because they made sex less pleasurable. Because of their disempowerment, many women therefore will not insist on a condom and give in to their partner’s desire to ‘taste the candy’. ^10^

## INTIMACY AND TRUST

‘At the start of the relationship we were playing, now I trust her, that is why I don’t use it.’^7^

(Zulu man)

One of the most important barriers to condom usage for women is the issue of intimacy and trust. The use of condoms is perceived to be associated with casual sex, and where there is ‘true love’ condoms are no longer used. In the standard progression of romantic relationships, after trust has been established, condoms are no longer perceived to be needed.^4^

‘He said he uses condoms with his seven sex buddies, but not with his girlfriend because he loves her.’

(Lay HIV counsellor, quoted from an Anglican  Students Fellowship Conference, Bellville, 2006)

In Reddy’s study of patients at STI clinics, 43% of men and 35% of women said that using a condom meant you do not trust your partner. People therefore associate condoms with casual sex and a lack of trust. 

Women’s self esteem and social status is often linked to a committed, monogamous relationship. In such circumstances suggesting condom use would be an insult – suggesting infidelity and a lack of ‘true love’. Condomless sex, on the other hand, helps maintain the desired image of the partner being faithful to them. So-called ‘unsafe sex’ is actually seen as keeping ‘safe’ the desired relationship and its intimacy, trust and economic stability. To acknowledge a possible infidelity and risk of HIV necessitates a confrontation, which may destabilise the relationship. A woman may also want to maintain the pretence that a casual encounter is actually a meaningful relationship. This means pretending to trust her partner, which may imply not using condoms and not questioning their sexual history. In this way, many people will make the choice to view themselves as ‘safe’ rather than face the social consequences of safer sex.^10^

## REPUTATION

‘My boyfriend says that if a man does not trust a woman, that is the only time to use condoms.’^11^

(Zulu woman)

AIDS has been presented as a disease with a high-risk of infection and condoms have become associated with the sexually promiscuous and with casual sex. Adolescents are primarily concerned with social acceptability and the opinions of their peers, so do not want to be associated with the negative connotations of condoms.^4^

Girls who suggest the use of condoms are considered, by both male and female participants, to be ‘loose’.^4^ A number of girls said they were not at risk for STIs and so had no need of condoms. Believing that you are at risk for AIDS would mean admitting that you have not been living up to certain standards.

Women feel embarrassment over every stage of condom use. When they are concerned for their reputation, then the act of buying condoms, carrying them and asking for their use is difficult. Having a condom on one’s person indicates a lack of sexual innocence, an unfeminine identity – that of a woman seeking sex too actively. A sexual woman becomes easy, fair game and generally at a man’s disposal.

To suggest condom usage may also give the impression that you are HIV positive. In South Africa and Uganda, wanting to use a condom can be interpreted as a sign that you are carrying disease.^12^ In Reddy’s study, 14% of men and 8% of women believed that ‘using a condom means that you have AIDS.’ 

‘I would be embarrassed and afraid. Maybe the guy would think I have AIDS then he wouldn’t want to have sex with me.’

(Zulu woman)^10^

## POOR COMMUNICATION

There are often very low levels of communication between people who are involved in intimate physical and sexual acts. In Varga’s study of pregnant girls and their partners, 61% of girls felt that AIDS-related issues were not appropriate to discuss with their partners. None of the males had discussed AIDS with the mothers of their children. Females focused on lack of intimacy as a reason for avoiding the discussion.

‘We don’t talk about things like condoms, sex or STDs [sexually transmitted diseases]. It isn’t that kind of relationship.’^10^

(Zulu woman)

With such poor levels of communication, it becomes nearly impossible to discuss condoms.

## FEAR OF REJECTION AND VIOLENCE

‘I would be afraid of his reaction. He might leave me.’

(Zulu woman)

Male opposition to contraceptives is common. A study from Soweto, Umlazi and Khayelitsha found that fear of losing a partner was the most important barrier to women’s contraceptive use.^13^ There is also the fear that rejection will lead to violence.^8,10^ In Pettifor’s study, women who experienced forced sex are 5.8 times more likely to use condoms inconsistently.^14^

‘I would not talk to my boyfriend about contraception. If he thought I was using it, he would beat me.’^10^

(Zulu woman)

## RELIGIOUS BELIEFS

In many religious communities, the condom is associated with immoral and sinful behaviour.

‘We are saved by the blood of the lamb, not by a piece of rubber.’

(Sermon at a Youth Congress, Anglican  Church, Khayelitsha, 2001)

‘How can you include this information on condoms? You are promoting fornication.’

(Inter-church Conference, Cape Town, 2004, when the information pack contained leaflets from the Department of Health indicating the correct way to put on condoms)

The President of the Vatican’s Pontifical Council for the Family made the following statement on *Catholic online*, suggesting that condoms should carry a government health warning:

‘The AIDS virus is roughly 450 times smaller than the spermatozoon. The sperm can easily pass through the ‘net’ formed by the condom. These margins of uncertainty should represent an obligation on the part of the health ministries and all these campaigns to act in the same way as they do with regard to cigarettes, which they state to be a danger.’^15^

  At one rally organised by a faith-based organisation in Uganda, participants were told that ‘using a condom with a person with these [sexually transmitted] diseases is like using a parachute which only opens 75% of the time.’ ^16^ ‘be wise, don’t condomise,’ was the message from a Catholic Publication in Nairobi called HIV/AIDS: A call to action: Responding as christians.^17^

## CONCLUSION

**FIGURE 1:**
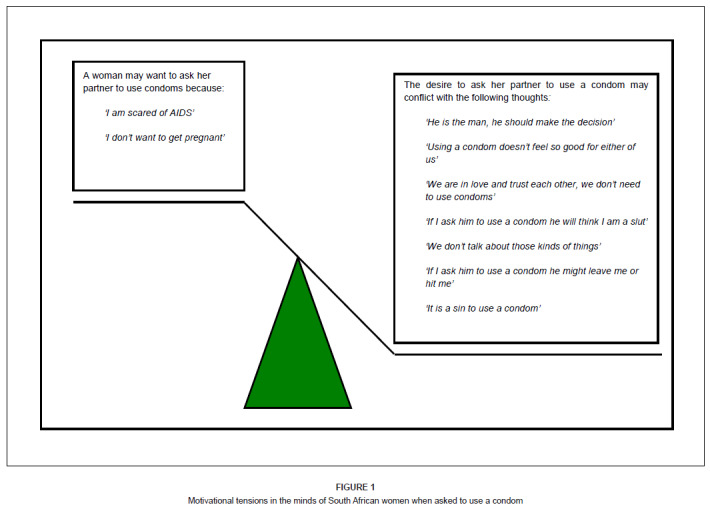
Motivational tensions in the minds of South African women when asked to use a condom

It is clear from this discussion that when a health worker advises a woman to use a condom, the decision for the woman is more complex than the health worker’s desire to prevent transmission of HIV. Most people, when faced with a behaviour change, such as using condoms, will feel ambivalent with internal arguments both for and against the change. The arguments for and against change can be likened to the weights on either end of a balance or scale. The literature on condoms, however, suggests that the arguments against change, which are largely relational and not medical, often outweigh the arguments for using condoms (Figure 1). Prevention workers who only acknowledge one side of the scale may find the process of arguing for condom use a frustrating one that is met with overt opposition, covert non-adherence or only superficial agreement. Arguing forcefully for condom use (one side of the balance) may perversely encourage the woman to argue the case for not using condoms (the other side of the balance) and even increase resistance to change. A more helpful approach may be to explore the pros and cons with the client and to enable her to find the solutions to overcome potential barriers, rather than to presume that the decision is simple and clear-cut. This implies that a guiding style rather than a directing style may be more effective and that health workers may need more effective communication skills when recommending the use of a condom.^18^

One resource could be Motivational Interviewing, which has been characterised as a refined form of a guiding communication style. This style of communication is primarily a way of interacting with patients that is at once collaborative, curious, respectful of their autonomy and evocative of the patients’ own perspectives and solutions. Key principles include the use of empathic active listening, reflection of discrepancy between the patients’ behaviour and personal values or goals, amplification of ‘change talk’ and reduction of ‘sustain talk’, information exchange and strengthening of self-efficacy. Health workers are sensitive in their approach to patients’ agenda and readiness to change, while focusing on a specific behaviour. Health workers aim to help the patients resolve their own ambivalence. Specific communication skills that can be learnt within this framework are, for example, the use of a variety of reflective listening statements, summaries and open questions.^18^

## FURTHER RESOURCES

MISA (Motivational Interviewing in Southern Africa) offers training in a guiding style and communication skills: http://www.sahealthinfo.org/motivational/index.htm. 
